# Choosing a Surgical Access Point for Hysterectomy: A Paradigm Shift Over a 10-Year Span

**DOI:** 10.3389/fmed.2020.569895

**Published:** 2020-11-25

**Authors:** Florian Ebner, Niko de Gregorio, Christiane Lato, Valerie Ohly, Wolfgang Janni, Jennifer Spohrs, Lucia Jerg-Bretzke, Steffen Walter

**Affiliations:** ^1^Department of Gynaecology, Helios Amper-Hospital Dachau, Dachau, Germany; ^2^Department of Gynaecology and Obstetrics, Ulm University Hospital, Ulm, Germany; ^3^Medical Psychology Division, Department of Psychosomatic Medicine and Psychotherapy, Ulm University Hospital, Ulm, Germany

**Keywords:** surgical access for hysterectomy, choosing the surgery access, duration of surgery, stay of hospital, uterus weight, age, BMI

## Abstract

**Background:** When choosing a surgical procedure for a hysterectomy, doctors and patients have various options in terms of the multiple surgical access points available. The aim of this study was to descriptively analyze developments concerning the surgical access point selected over the past 10 years at Ulm University Hospital, (south) Germany, assess the variables associated with the surgical method and explore any potential significant correlations that influence these surgical access routes. Explicitly, we wished to investigate whether the approval of ulipristal acetate and the warning issued by the Food and Drug Administration (FDA) in connection with its use changed existing trends.

**Material and Methods:** This monocentric study retrospectively assessed data from all patients who underwent a hysterectomy due to a benign disease or endometrial cancer from January 2007 until December 2016.

**Results:** Of the benign indications considered, myomas and descensus genitalis occurred most frequently (49.5 and 30.6%, respectively). The percentage of abdominal procedures declined from 61.4 to 13.4% between 2007 and 2016 for all hysterectomies, whilst it increased from 4.1 to 69.7% for laparoscopic hysterectomies. The rate of vaginal hysterectomies increased to 45.5% until 2013 and declined in the years afterwards. Laparoscopic assisted vaginal hysterectomies were comparatively rare.

The trends in terms of surgical routes were similar for endometrial cancer. During the observation period, the share of abdominal hysterectomies fell from 100 to 11.3%, whilst the share of laparoscopic hysterectomies increased from 0 to 86.6%. The other two procedures were less frequently used.

Use of the laparoscopic hysterectomy procedure also increased significantly after the FDA's 2014 warning. Ulipristal acetate may have tended to influence the process.

**Conclusion:** Contrary to the national decrease in hysterectomy numbers, the annual number of hysterectomies at Ulm University Hospital remained stable during the observation period. Nevertheless, there was a clear shift in the preferred surgical routes for hysterectomy.

## Introduction

A hysterectomy (removal of the uterus) is the second most frequent surgical procedure in the field of gynecology after a Cesarean section (1, 2). The number of hysterectomies being performed has decreased both internationally (3, 4) and nationally. For example, 111,673 hysterectomies were conducted in Germany in 2016 (2), which represents a decline of nearly 45,000 hysterectomies compared to 2007 (5). With a prevalence of 17.5%, approximately every sixth woman between the ages of 18 and 79 undergoes this procedure (6). The highest incidence was 48% for women from the age of 40 to 49 in 2012. The reasons for a hysterectomy are manifold and encompass benign as well as malignant diseases. Benign diseases represent the majority of indications for a hysterectomy (6), with numbers varying from 75.6 to 90%, depending on the study (3, 7). There are multiple surgical methods for removing the uterus, allowing surgeons to select the procedure and access route that best suit the individual patient based on their general state of health. The choice of surgical procedure depends on various factors: size and shape of the uterus and vagina, mobility of the uterus, preceding surgeries, comorbidities of the patient, necessity of the execution of a parallel extrauterine procedure, local surgery conditions, infrastructure of the hospital, uterus type, level of experience of the surgeon, requests of the patient, and the question of whether it is a planned or an emergency procedure (3, 8). In 2011, Park et al. conducted a critical discussion of the morcellation of the uterus (9). Subsequently, the Food and Drug Administration (FDA) released a warning concerning electrical morcellation 2014 (10). The S3 Guidelines on Hysterectomy published in Germany by the Association of the Scientific Medical Societies in Germany (Arbeitsgemeinschaft der Wissenschaftlichen Medizinischen Fachgesellschaften, AWMF) suggest the use of vaginal access instead of laparoscopic or open aditus. The German Robert Koch-Institut Berlin published national data on hysterectomy in 2014 (11, 12). In March 2012, ulipristal acetate was approved in Germany with the aim of reducing the growth of benign tumors followed by an FDA warning on power morcellation in 2014 (9). Whilst this had a major impact on the surgical route in the USA, this has not been reflected in the numbers for European hospitals.

### Research Question and Aim of This Work

The aim of this retrospective study was to assess the trends in the multiple surgical procedures for hysterectomy (Abdominal Hysterectomies: AH; Vaginal Hysterectomies: VH; Laparoscopic Assisted Vaginal Hysterectomies: LAVH; Laparoscopic Hysterectomies: LH) during the past 10 years at Ulm University Hospital, The work addresses the following questions:

- Did any changes occur in the surgical route in the decade (2007–2016)?
- Did any such changes influence the time a patient spent in theater and the length of hospitali-zation?
- Did the approval of ulipristal acetate and the FDA warning change pre-existing trends?
- Did the introduction of ulipristal acetate have an impact on the weight of the uterus?
- Did the uterine weight, age, and BMI score of the patient affect the choice of surgical route?

## Methods

### Data Acquisition

Data acquisition for the patient collective took place in a retrospective manner using the electronically stored medical records in the SAP system from February to September 2017. The timeframe set for data collection was 1st January, 2007 to 31st December, 2016. During this decade, there were no adjustments related to the electronical documentation and no changes in the integration of relevant subsystems. The surgery indication and diagnosis of the patient were derived from the ICD-10 codes encoded and stored in the SAP system. Furthermore, the last laboratory value prior to surgery and the first laboratory value post-surgery were electronically assessed from the laboratory data bank (as well as additional variables), but this will not be mentioned further in this study.

### Inclusion/Exclusion Criteria

All patients with an ICD code for a hysterectomy in the aforementioned timeframe were included in the study.

Patients were identified by means of the documented OPS (Operation and Procedure Classification System) codes: 5-682 (subtotal uterus extirpation), 5-683 (uterus extirpation [hysterectomy] with or without salpingo-oophorectomy), or 5-685 (radical uterus extirpation with or without salpingooophorectomy as well as mesometrial resection of the uterus). Patients with a cervical stump resection (OPS 5-684) and patients who underwent a hysterectomy as part of an oncological surgery were excluded with the exception of patients with a primary diagnosis of endometrial carcinoma. The inclusion of patients with an endometrial carcinoma was supported by the homogeneity of the surgery and the discussion regarding the ideal surgical access method.

Further exclusion criteria included the unpredicted change of the surgical access route, except for the planned LAVH. Reasons for an unexpected change of surgical access may come from the patient's side (abdominal adhesions etc.), the surgeon's side (laparoscopically uncontrollable bleeding, unclear situs etc.), or may be linked to anaesthesiological factors (respiratory problems, etc.).

### Statistical Analyses

The descriptive statistical analysis was performed by indicating the absolute and relative frequencies. Since the distribution of the metric variables analyzed varied significantly from the normal distribution (Shapiro-Wilk-Test), only non-parametric statistical analyses were conducted. IBM SPSS statistics version 25 was used for the analyses (Spearman Correlation, Kruskal–Wallis and *U*-tests). The significance level was *p* < 0.05.

## Results

**Figure 1:**
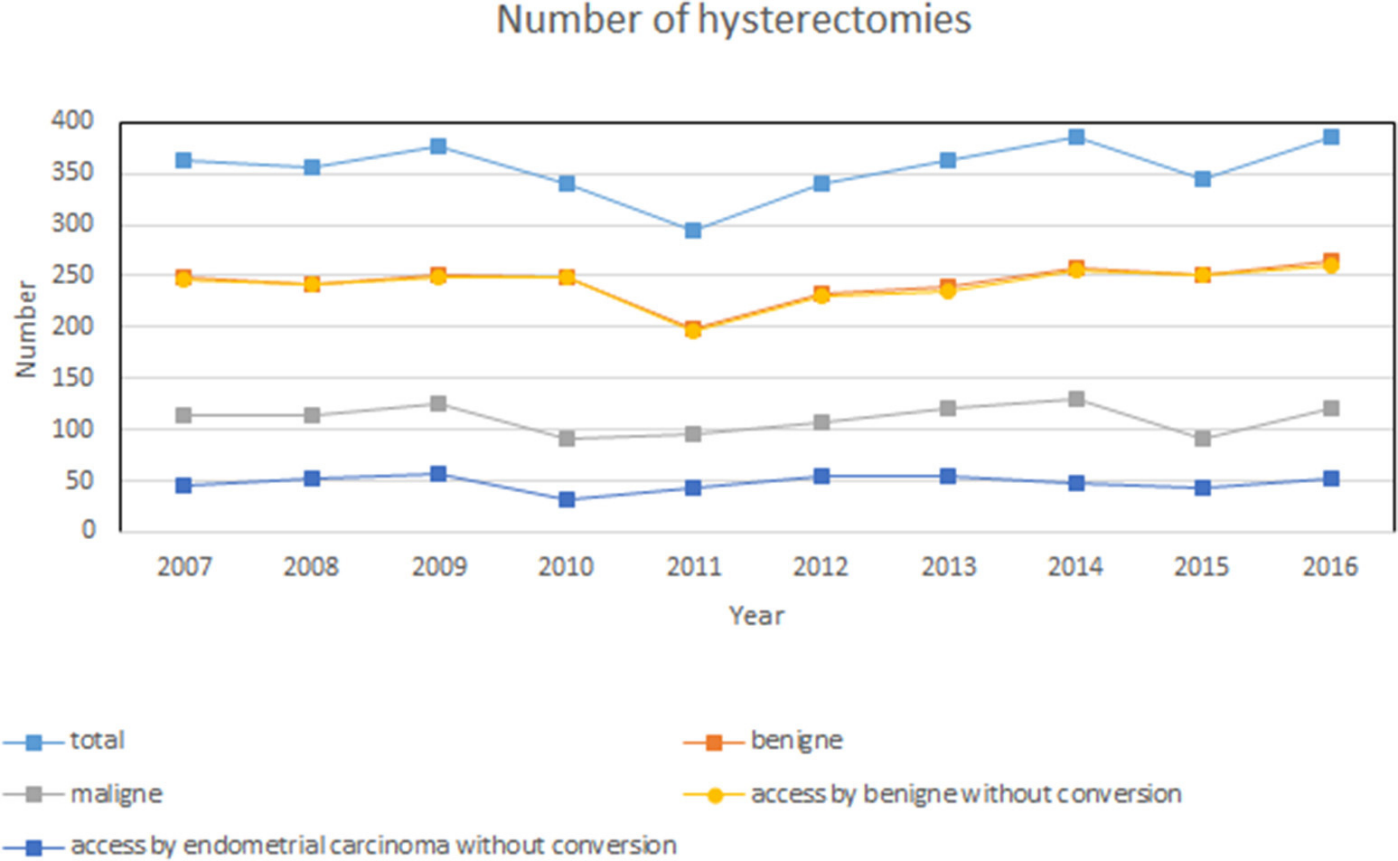
Number of yearly surgeries during the period assessed from 2007 to 2016. Sum of all 3,545 hysterectomies. Inclusion and exclusion criteria for the present study: 2,415 benign data sets (access without conversion) and 483 data sets (access without conversion) with endometrial carcinoma.

During the observation period, 3,545 hysterectomies were documented in the hospital's information system. On average, a total of 354.5 ± 27.4 hysterectomies were conducted every year (min. 294 in 2011, max. 386 in 2014 and 2016) (Figure 1). Based on the inclusion and exclusion criteria, 2,415 benign data sets (access without conversion) were analyzed and 483 (access without conversion) sets for patients with an endometrial carcinoma. The total number of patients analyzed was 2,898.

The most common indications for benign hysterectomies were myomas (49.5%), descensus (30.6%) and bleeding complaints (21.9%), followed by endometrioses (6.6%) and cervical dysplasia (4.8%). The percentage of patients with diseases that did not fit into any of these categories was 16.6%.

### Hysterectomy for Benign Diseases and Endometrial Carcinoma Over the Course of the Observation Period (2007–2016)

#### Choosing the Surgical Route

**Figure 2:**
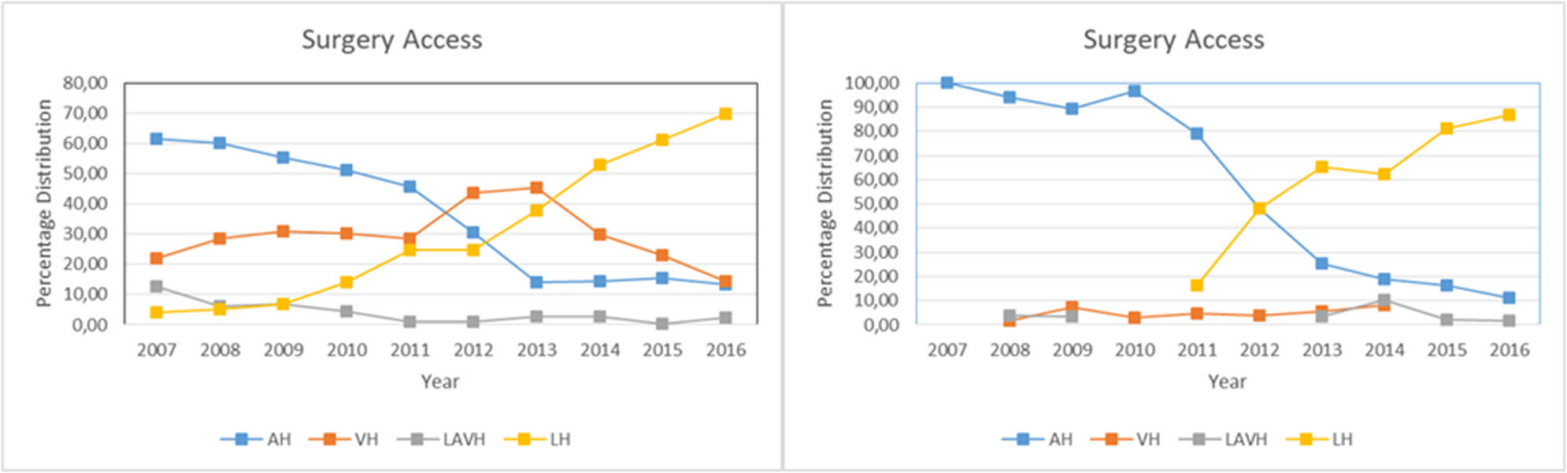
Choice of surgical access route. **(Right)** Hysterectomies without conversion for benign diseases: *N* = 2,415; AH, abdominal hysterectomies: *N* = 866; VH, vaginal hysterectomies: *N* = 711; LAVH, laparoscopic assisted vaginal hysterectomies: *N* = 98; LH, laparoscopic hysterectomies: *N* = 740. **(Left)** Hysterectomies without conversion for endometrial carcinoma: *N* = 483; AH: *N* = 273, VH: *N* = 17, LAVH: *N* = 13, LH: *N* = 180.

Figure 2 (left) shows the surgical access for hysterectomies in women with benign diseases. AH decreased from 61.4% in 2007 to 13.4% in 2016. For LH, the opposite was the case, and the percentage of these procedures increased from 4.1 to 69.7% over the 10-year span. For VH (21% in 2007) an increase to 45.5% was observed until 2013, which then decreased to 14.6% in 2016. Meanwhile, a decline from 12.6 to 2.3% was recorded for LAVH.

Contrary to the assumption that the LH numbers would decrease following the FDA warning in 2014, the data shows an increase in LH procedures until 2016. The increase rate before the FDA warning was issued (pre-FDA: April 2013–March 2014) vs. afterwards (post FDA: May 2014–April 2015) is highly significant (*p* < 0.001).

The uterine weight can be used to support the decision concerning the surgical route.

The average annual uterine weight was as follows: in *2007*: M = 313.91 g ± 490.69; *2008*: M = 272.87 g ± 546.31; *2009*: M = 292.54 g ± 461.46; *2010*: M = 288.59 g ± 428.98; *2011*: M = 326.32 g ± 444.81; *2012*: M = 248.96 g ± 399.34; *2013*: M = 273.43 g ± 540.59; *2014*: M = 209.86 g ± 238.69; *2015*: M = 271.26 g ± 444.20; *2016*: M = 247.94 g ± 339.64.

Comparing the average uterine weight in the period from January 2007 to February 2012 (pre ulipristal acetate: M = 293.21 g ± 473.13) vs. April 2012–December 2016 (post ulipristal acetate: M = 251.30 g ± 402.35), there is a significant decrease in uterus size (*p* < 0.004). As the use of ulipristal acetate may have increased over time, the uterine weight analysis was also conducted for the period March 2011–February 2012 (pre ulipristal acetate approval: M = 309.82 g ± 442.66) vs. April 2012–March 2013 (post ulipristal acetate approval: M = 255.97 g ± 408.21) and showed a significant (*p* < 0.032) difference in uterine weight.

From 2011 to 2012, the procedure of the LH stagnated (Figure 1, left), which significantly (*p* < 0.001) increases again after 2012.

Figure 2 (right) shows the endometrial carcinoma group during the observation period. Similarly to the group with benign diseases, this group experienced a steady decline in AH procedures (100–>12%) and a significant increase in LH procedures (17–>87%).

Contrary to the benign disease group, there was no significant change for LH in the endometrial carcinoma group immediately after the FDA warning was issued (pre FDA: April 2013–March 2014) vs. afterwards (post: May 2014–April 2015). Instead, a significant change was observed after a year [post FDA (1 year later): May 2015–April 2016] (*p* < 0.007).

#### Correlation for Diseases (Benign and Endometrial Carcinoma) and Duration of Surgery and Hospital Stay

A Spearman correlation shows a significant decrease in the *duration of surgery* over the years for all surgical techniques in the group of patients with benign diseases but this was only considered in detail for AH and LH.

The endometrium carcinoma group also shows a significant decrease in the duration of surgery across the board, but a significant decrease was only calculated for LH (see Table 1, left half) when all procedures were considered separately.

**Table 1:** Connection (Spearman correlation) between the time course (2007–2016) of surgery duration and hospital stay (AH, abdominal hysterectomies; VH, vaginal hysterectomies; LAVH, laparoscopic assisted vaginal hysterectomies; LH, laparoscopic hysterectomies).

	**Benign diseases**					
**Surgery access**	**Duration of surgery (min)**	**Correlation (r)**	***p*-value**	**Duration of hospital stay (day)**	**Correlation (r)**	***p*-value**
AH (*N* = 866)	138 ± 66	−0.215	0.001	6.9 ± 2.8	−0.332	0.001
VH (*N* = 711)	69 ± 37	0.145	0.001	4.3 ± 1.3	−0.313	0.001
LAVH (*N* = 98)	145 ± 51	−0.02	n.s.	5.5 ± 4.1	−0.253	0.012
LH (*N* = 740)	113 ± 48	−0.191	0.001	3.6 ± 1.7	−0.310	0.001
**Total (*****N*** **=** **2,415)**	**110.3** **±** **60.3**	**−0.152**	**0.001**	**5.1** **±** **2.7**	**−0.524**	**0.001**
	**Endometrial carcinoma diseases**					
**Surgery access**	**Duration of surgery (min)**	**Correlation (r)**	***p*****-value**	**Duration of hospital stay**	**Correlation (r)**	***p*****-value**
AH (*N* =273)	224 ± 119	0.031	n.s.	11 ± 4.7	0.08	n.s.
VH (*N* =17)	86 ± 32	−0.105	n.s.	5.4 ± 1.6	−0.214	n.s.
LAVH (*N* = 13)	128 ± 48	−0.162	n.s.	4.8 ± 1.2	−0.417	n.s.
LH (*N* =180)	110 ± 64	−0.400	0.001	5.3 ± 5.6	−0.325	0.001
**Total (*****N*** **=** **483)**	**173.7** **±** **113.8**	**−0.414**	**0.001**	**8.5** **±** **5.7**	**−0.502**	**0.001**

The length of *hospital stay* has decreased significantly over the last 10 years both in general and for each separate surgical access method in the benign diseases group.

A significant general decrease in hospitalization time was also observed for the endometrial carcinoma group. When the various surgical access routes were considered individually, however, a significant decrease was only observed for LH (see Table 1, right half).

For both groups—benign diagnosis and endometrial carcinoma (Table 1)—the analysis shows a significant difference in both the *duration of the surgery* (*p* < 0.001) and the *hospitalization period* (*p* < 0.001). In the benign disease group, the longest surgery durations were significantly associated with LAVH vs. the other surgical routes (*p* < 0.001), whereas in the endometrial carcinoma group, surgery duration for AH was significantly longer than for the other surgical routes (*p* < 0.001). The longest hospital stay was associated with AH vs. the other access points (*p* < 0.001) regardless of the indication. Discharge was significantly faster for patients receiving LH vs. the other access routes (*p* < 0.001).

### Use of Hysterectomy for Benign Diseases and Endometrial Carcinoma Depending on Duration of Surgery, Hospital Stay and Age, BMI, Uterus Weight, and Drainage Flow Rate

Table 2 presents a descriptive differentiation of the possible approach in terms of age, BMI, uterine weight, and drainage flow rate.

**Table 2:** Descriptive differentiation of the surgical approach in terms of age, BMI, uterine weight (UW), and drainage flow rate (DFR).

		**Benign diseases**				**Endometrial carcinomas**			
**Surgery access**		**Age (years)**	**BMI (kg/m^**2)**^**	**UW (g)**	**DFR (ml)**	**Age (years)**	**BMI (kg/m^**2**^)**	**UW (g)**	**DFR (ml)**
AH	M	49.48	26.77	465.84	381.67	65.95	29.13	164.48	1977.36
	SD	8.85	5.8	645.37	637.84	10.09	7.39	282.11	2915.86
VH	M	59.12	26.36	87.55	241.11	74.24	31.59	84.14	52.5
	SD	12.51	4.67	58.28	376.98	9.05	8.36	31.55	24.75
LAVH	M	48.65	26.79	182.02	302.68	68.38	32.79	109.91	132.5
	SD	9.18	6.42	117.95	427.66	10.3	9.4	74.75	133.50
LH	M	48.03	27.29	217.22	261.8	64.39	29.72	109.1	461.47
	SD	8.68	5.83	176.76	271.01	10.4	8.17	57.68	1519.89
Kruskal-Wallis-Test	p-level	0.001	*n.s*.	0.001	0.001	0.001	*n.s*.	*n.s*.	0.001

Older patients underwent VH significantly (*p* < 0.001) more frequently for both benign and malignant diagnoses.

No significant difference in surgical methods was found in connection with the *BMI* of the patients in either of the diagnosis groups.

The analysis of the *uterus weight* yielded significant differences (*p* < 0.001) between the four surgical methods. The heaviest uteruses were removed by means of AH (*p* < 0.001). Contrary to the benign groups, the uterine weight did not vary significantly in patients with malignant diagnoses.

AH shows a significant *drainage flow rate* compared to minimally invasive procedures for both groups (*p* < 0.001).

## Discussion

The number of hysterectomies performed at Ulm University Hospital remained constant from 2007 to 2016 even though the number of hysterectomies in Germany declined during this period, which is also documented in the regional analysis of the Ärzteblatt German-language medical magazine (4, 13). The politically motivated closing of smaller gynecological departments led to a centralization of surgeries in larger centers within the country (14). The detailed analysis shows a clear shift in the choice of the surgical access route for hysterectomy. The percentage of minimally invasive procedures increased for both patients with benign diseases and those with endometrial carcinoma, while the percentage of open surgeries decreased.

The advantages of minimally invasive procedures for hysterectomies have been outlined in prospective studies with large samples and different points of view. These include a faster convalescence, a quicker return to work and lower costs for the procedure (3, 15).

The transition from open to minimally invasive surgery has been documented in various countries (16, 17). Although our analysis confirms the published data, two events occurred during our observation that should have changed the clinical management, namely the approval of ulipristal acetate in 2012 and the issuing of the FDA warning in 2014. Both events could have stopped the transition toward minimally invasive surgery. Our data shows that this change began to occur in patients with benign diagnoses in 2009/2010 and in those with endometrial carcinoma in 2011. At the same time, Park et al. published a retrospective analysis on sarcomas of the uterus. For this relatively rare disease, the authors present a deterioration of the prognosis for one-third of the patients who received a morcellation of the leiomyoma sarcoma in a large sample with a long observation period. This and similar publications prompted the FDA to issue a warning about morcellation and as part of this controversy, the minimally invasive procedure was put up for discussion (18). The topic was also debated at Ulm University Hospital. The present analysis shows that this open-ended discussion did not lastingly influence the trend toward minimally invasive procedures (19, 20). This result is remarkable since efforts to achieve a reliable preoperative sarcoma diagnosis have not yet yielded a feasible test (13, 21) and Desai et al. (20), for example, report a clear decline in supracervical hysterectomies in the wake of the FDA warning. The approval of ulipristal acetate may even have increased the rates of LH, as analysis has shown a significant reduction in uterine weight after 2012. In view of this, it would be interesting to investigate whether the use of LH declined once again in Germany after 2018.

BMI and uterine weight have also been addressed by other authors as decision criteria (22–24). A retrospective data analysis by Tyan et al. (23) of nearly 160,000 data sets showed a positive correlation between BMI and the development of post-surgery complications in patients receiving open surgery as compared to those receiving laparoscopy. This indicates that obese patients pose a challenge for many surgeons and should only undergo surgery at centers with the necessary expertise. Our data does not confirm these findings. However, this might be due to the high surgical standards and the expertise in bariatric surgery at Ulm University.

Whilst at the beginning of the decade, a drain was inserted at every surgery to remove excessive wound serum, this rate decreased, especially for minimally invasive procedures. Our analysis shows a clear difference in serum production between patients receiving LH and those receiving AH. This is in line with previously published observations of a speedier recovery after surgery (25). Furthermore (preoperative) recommendations to conduct outpatient hysterectomies in a subgroup of patients are increasing. In addition to careful patient selection and preparation, these recommendations exclude the insertion of a drain (15, 26). Further differences noted include the duration of the abdominal hysterectomy in benign and malignant cases. This difference could be due to the varying types of incision (horizontal vs. longitudinal), BMI (26.7 vs. 29.3 kg/m^2^), the weight of the uterus (466 vs. 164 g), or the patient's age (49.5 vs. 65.9 years). Further research is warranted in this area with a larger sample size.

As a result of this discussion the emphasis on “informed consent” was even stronger. Education according to the guidelines was implemented in the team at an early stage (27). Morcellation bags were not used during the period of investigation as these were still under development, and no safety data was available. Preoperatively suspected malignancies or patients who expressed a wish for the treatment with the lowest oncological risk underwent surgery without morcellation. These patients were offered the option to participate in the study on preoperative Vascular Endothelial Growth Factor **(**VEGF) determination (21) and documented in a nationwide registry study. Further biomarkers and their limitations, as described by Ogawa et al. (28) were discussed with the patients.

The result of the informed consent analysis showed that some patients opted for the faster recovery and others preferred a lower oncological risk despite a potentially higher surgical risk of additional organ injuries.

A further longitudinal study is to be conducted to determine the extent to which the course of surgical access will change or stabilize in the future, both nationally and internationally.

## Conclusion

Despite various events during the study period, the trends in the surgical route for Ulm hospital (southern Germany) continued to shift from open surgery to minimally invasive surgery even after the FDA warning in 2014. The surgical route was significantly associated with uterine weight (Explicitly in the procedure of the AH). The benefits of minimally invasive surgery are visible over a long period.

## Data Availability Statement

The datasets presented in this article are not readily available due to German data protection regulations. Requests to access the datasets should be directed to florian.ebner@hellos-gesundheit.de.

## Ethics Statement

The studies involving human participants were reviewed and approved by Ethics Commission Ulm University, Germany (Number of Votum: 253/17). The patients/participants provided their written informed consent to participate in this study.

## Author Contributions

FE: literature search, data analysis, and writing. ND: study design and writing. CL, LJ-B, and JS: critical revision. VO: data collection and data analysis. WJ: critical review. SW: critical revision, data interpretation, and writing. All authors: contributed to the article and approved the submitted version.

## Conflict of Interest

The authors declare that the research was conducted in the absence of any commercial or financial relationships that could be construed as a potential conflict of interest.

## Abbreviations

AH: Abdominal Hysterectomies
AWMF: Association of the Scientific Medical Societies in Germany/Arbeitsgemeinschaft der Wissenschaftlichen Medizinischen Fachgesellschaften
BMI: Body Mass Index
FDA: Food and Drug Administration
ICD-10: International Classification of Diseases
LH: Laparoscopic Hysterectomies
LAVH: Laparoscopic Assisted Vaginal Hysterectomies
OPS: Operation and Procedure Classification System
SAP, Systeme: Anwendungen und Produkte in der Datenverarbeitung
VH: Vaginal Hysterectomies.
